# Resistive switching memory characteristics of Ge/GeO_*x*_ nanowires and evidence of oxygen ion migration

**DOI:** 10.1186/1556-276X-8-220

**Published:** 2013-05-08

**Authors:** Amit Prakash, Siddheswar Maikap, Sheikh Ziaur Rahaman, Sandip Majumdar, Santanu Manna, Samit K Ray

**Affiliations:** 1 Department of Electronic Engineering, Chang Gung University, 259 Wen-Hwa 1st Rd., Kwei-Shan, Tao-Yuan 333, Taiwan; 2 Department of Physics and Meteorology, Indian Institute of Technology, Kharagpur, West Bengal 721302, India

**Keywords:** RRAM, Ge/GeO_*x*_, Nanowire, Nanofilament, Oxygen ion migration, Memory

## Abstract

The resistive switching memory of Ge nanowires (NWs) in an IrO_*x*_/Al_2_O_3_/Ge NWs/SiO_2_/p-Si structure is investigated. Ge NWs with an average diameter of approximately 100 nm are grown by the vapor–liquid-solid technique. The core-shell structure of the Ge/GeO_*x*_ NWs is confirmed by both scanning electron microscopy and high-resolution transmission electron microscopy. Defects in the Ge/GeO_*x*_ NWs are observed by X-ray photoelectron spectroscopy. Broad photoluminescence spectra from 10 to 300 K are observed because of defects in the Ge/GeO_*x*_ NWs, which are also useful for nanoscale resistive switching memory. The resistive switching mechanism in an IrO_*x*_/GeO_*x*_/W structure involves migration of oxygen ions under external bias, which is also confirmed by real-time observation of the surface of the device. The porous IrO_*x*_ top electrode readily allows the evolved O_2_ gas to escape from the device. The annealed device has a low operating voltage (<4 V), low RESET current (approximately 22 μA), large resistance ratio (>10^3^), long pulse read endurance of >10^5^ cycles, and good data retention of >10^4^ s. Its performance is better than that of the as-deposited device because the GeO_*x*_ film in the annealed device contains more oxygen vacancies. Under SET operation, Ge/GeO_*x*_ nanofilaments (or NWs) form in the GeO_*x*_ film. The diameter of the conducting nanofilament is approximately 40 nm, which is calculated using a new method.

## Background

One-dimensional semiconductor nanostructures such as nanotubes and nanowires (NWs) are being actively investigated for applications in electronic, photonic, and sensor devices [1]. Group IV semiconductor NW-based devices are attractive because of their compatibility with the existing Si complementary metal oxide semiconductor (CMOS) integrated circuit technology. Therefore, group IV NWs such as Ge/GeO_*x*_ can also be used for nanoscale nonvolatile memory applications because they are compatible with CMOS technology. Resistive random access memory (RRAM) devices have received considerable interest recently because of their high performance and potential scalability [2-8]. In recent years, many solid electrolyte materials such as Ge_*x*_Se_1 − *x*_[9-12], GeS_2_[13], Ta_2_O_5_[14], Ag_2_S [15,16], ZrO_2_[17], TiO_*x*_/ZrO_2_[18], GeSe_*x*_/TaO_*x*_[19], HfO_2_[20], CuTe/Al_2_O_3_[21], and Ti/TaO_*x*_[22] have been used in conductive bridging random access memory (CBRAM) applications. RRAM devices containing materials such as HfO_*x*_[5,6], SrTiO_3_[7], TiO_2_[8,23], ZrO_2_[24,25], Na_0.5_Bi_0.5_TiO_3_[26], NiO_*x*_[27], ZnO [28,29], TaO_*x*_[30,31], and AlO_*x*_[32,33] have been reported. However, GeO_*x*_ has only been used in RRAM as Ni/GeO_*x*_/SrTiO_*x*_/TaN [34] and Cu/GeO_*x*_/W [35] structures and in Ge-doped HfO_2_ films [36]. RRAM devices containing nanotubes and Si NWs have also been reported [37-39]. Although many switching materials and structures have been developed, the switching mechanism of RRAM devices remains unclear despite it being very important for application of RRAM. Ge/GeO_*x*_ NWs in an IrO_*x*_/Al_2_O_3_/Ge NWs/SiO_2_/p-Si metal oxide semiconductor (MOS) structure have not been reported either. Because of the self-limitation of current compliance (CC < 20 μA) in MOS structures, here we fabricate an IrO_*x*_/GeO_*x*_/W metal-insulator-metal (MIM) structure to understand how the resistive switching mechanism involves oxygen ion migration through the porous IrO_*x*_ electrode. It is also important to investigate the scalability potential of RRAM devices. The size of devices is typically limited by equipment or cost, so the diameter of conducting pathways could be investigated using switching characteristics or leaky pathways rather than by fabricating large-scale devices. We believe the feature size of RRAM devices and their scalability potential will be considered the same as the diameter of the minimum conduction path in the future. We previously investigated the effect of nanofilament diameter on the properties of CBRAM devices [12]. However, a method to investigate the diameter of conducting paths in RRAM devices has not been developed. In this work, we determine the diameter of Ge/GeO_*x*_ nanofilaments in a GeO_*x*_ film within a MIM structure under SET operation using a new method. The results suggest that Ge/GeO_*x*_ NWs form under SET operation in the GeO_*x*_ film.

In this study, the growth of Ge NWs using the vapor–liquid-solid (VLS) technique is investigated. The fabricated core-shell Ge/GeO_*x*_ NWs are characterized by field emission scanning electron microscopy and high-resolution transmission electron microscopy. Defects in the Ge/GeO_*x*_ NWs are observed by X-ray photoelectron spectroscopy (XPS) and photoluminescence (PL) spectroscopy at 10 to 300 K. The resistive switching memory of the Ge/GeO_*x*_ NWs in an IrO_*x*_/Al_2_O_3_/Ge NWs/p-Si structure with a self-limited low current of <20 μA is determined. The mechanism of resistive switching involves oxygen ion migration, which is observed by the evolution of oxygen gas on the top electrode (TE) in an IrO_*x*_/GeO_*x*_/W structure under sufficient applied voltage. A device exposed to post-metal annealing (PMA) exhibits low-voltage operation (<4 V), low reset current (approximately 22 μA), and better data retention of >10^4^ s with large resistance ratio (>10^3^) than that of the as-deposited device, which is related to an increased number of oxygen vacancies in the GeO_*x*_ film. In addition, the diameter of the Ge/GeO_*x*_ nanofilaments (or NWs) of approximately 40 nm is calculated using a new method under SET. The low-current operation of this RRAM device will make it useful in nanoscale nonvolatile memory applications.

## Methods

**Figure 1:**
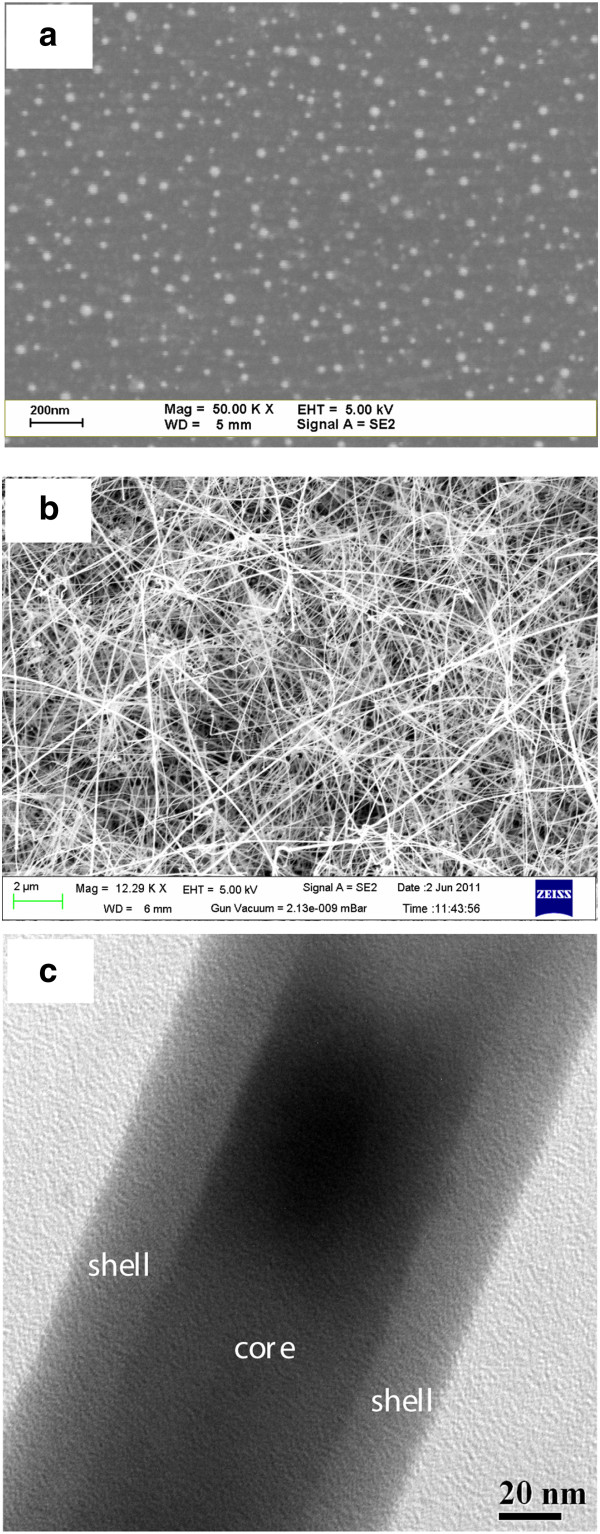
**SEM and TEM images.** SEM images of (**a**) Au nanoparticles and (**b**) Ge NWs on Si substrates. (**c**) TEM image of core-shell Ge/GeO_*x *_NWs.

**Figure 2:**
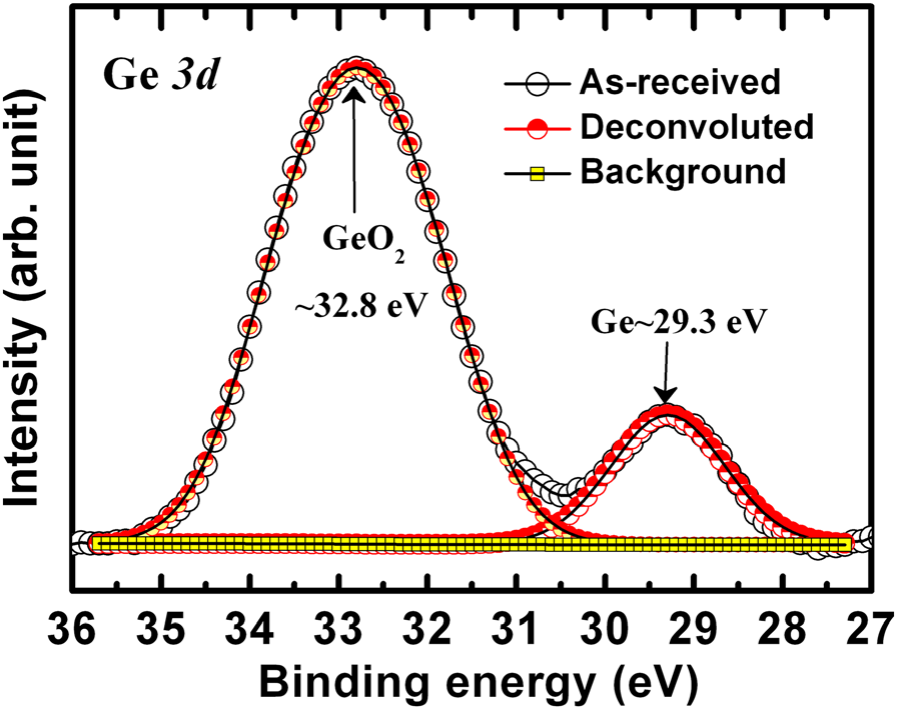
**XPS spectra of Ge 3*d *core-level electrons of the Ge/GeO**_***x ***_**NWs.**

**Figure 3:**
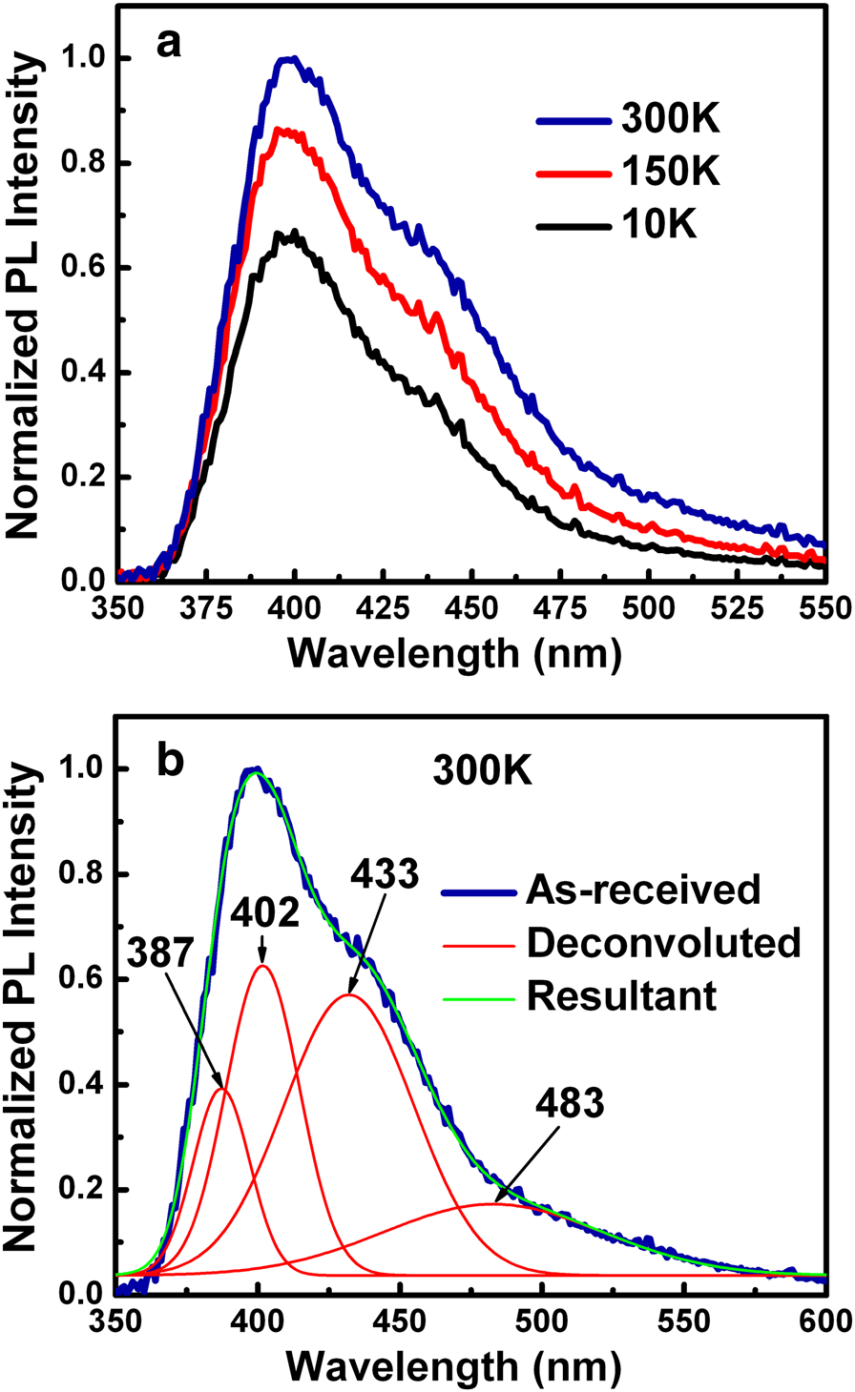
**PL and deconvoluted spectra.** PL spectra of the Ge/GeO_*x *_NWs (**a**) measured at temperatures of 10 to 300 K and (**b**) deconvoluted spectra at 300 K.

Ge NWs were grown by the VLS technique using Ge powder as the starting material (purity of 99.999%). Silicon (Si) wafers with an ultrathin gold (Au) coating as a catalyst were used as substrates. The substrate was annealed at 600°C for 30 min in a vacuum chamber to form isolated Au nanoparticles (NPs), or commercial Au NPs were used as substrates to grow NWs. The typical diameter of the Au NPs was approximately 5 nm, which was determined by scanning electron microscopy (SEM) (Figure 1a). Ge powder was placed in an alumina boat and inserted in a horizontal tube furnace. The furnace was heated at 900°C for 30 min under argon with a flow rate of 10 sccm to grow NWs through the VLS technique. High-density Ge NWs with a diameter of approximately 100 nm and length of approximately 100 μm were observed by SEM (Figure 1b). The Ge NWs possessed a core-shell structure, as shown in the transmission electron microscopy (TEM) image in Figure 1c. This suggests that the core region is Ge-rich, and the shell region is oxygen-rich, i.e., GeO_*x*_. It is expected that the GeO_*x*_ layer will contain more defects than the Ge-rich core, which may be useful for resistive switching memory applications. The defects in the Ge/GeO_*x*_ NWs were observed by both XPS and PL (Figures 2 and 3). PL measurements were obtained on a Triax 320 monochromator (Jobin Yvon, Edison, NJ, USA) and photomultiplier detector with an excitation wavelength of 325 nm.

**Figure 4:**
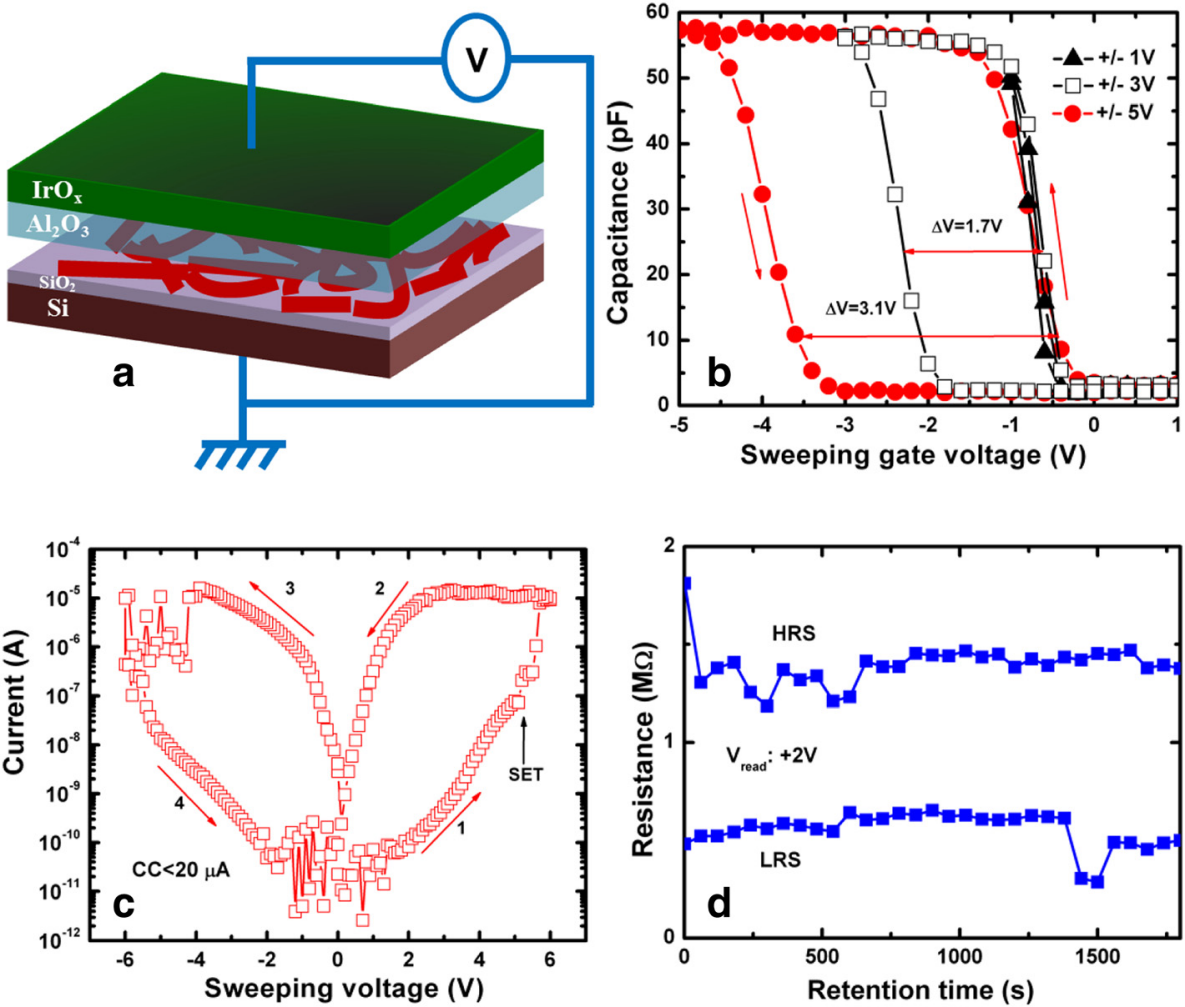
**Schematic diagram, charge-trapping phenomena, and typical *I*-*V *hysteresis and retention characteristics.** (**a**) Schematic diagram of the IrO_*x*_/Al_2_O_3_/Ge NWs/SiO_2_/p-Si MOS structure. (**b**) Charge-trapping phenomena observed by *C*-*V* measurements, proving the core-shell Ge/GeO_*x*_ nanowires to contain defects. (**c**) Typical *I*-*V* hysteresis characteristics of the resistive switching memory device with a MOS structure. A low CC of <20 μA is needed to operate this RRAM device. (**d**) Retention characteristics of the device.

Defects in the Ge/GeO_*x*_ NWs and resistive switching memory characteristics were also assessed by fabricating an IrO_*x*_/Al_2_O_3_/Ge NWs/SiO_2_/Si MOS structure, as shown in Figure 4a. MOS capacitors were fabricated using a shadow mask to pattern IrO_*x*_ electrodes onto Al_2_O_3_ that was grown on dispersed Ge/GeO_*x*_ NWs. The memory device consisted of three stacked layers: a top tunneling layer of Al_2_O_3_ (10 nm), a defect-rich Ge NW layer, and a thin tunneling layer of SiO_2_ (approximately 4 nm). After cleaning the Si wafer, an SiO_2_ layer was grown by annealing in a hot furnace as described above. The Ge/GeO_*x*_ NWs were then dispersed on the SiO_2_/Si substrate. To deposit the TE of IrO_*x*_, a thin layer of Al_2_O_3_ was also deposited. Both the Al_2_O_3_ and IrO_*x*_ layers were deposited by reactive radio-frequency (rf) sputtering. The vacuum of the chamber was approximately 2 × 10^−5^ Torr. An Al_2_O_3_ target was used to deposit the Al_2_O_3_ layer. The deposition power and chamber pressure were 80 W and 30 mTorr, respectively. The flow rates of Ar and O_2_ gas were 24 and 1 sccm, respectively, during film deposition. Finally, an IrO_*x*_ metal electrode with a nominal thickness of approximately 100 nm was deposited by rf sputtering using a shadow mask with a circular area of 3.14 × 10^−4^ cm^2^. An Ir target was used to deposit the IrO_*x*_ electrode, with a ratio of Ar to O_2_ gas of 1 (i.e., 25:25 sccm). The deposition power and chamber pressure were 50 W and 20 mTorr, respectively. The memory characteristics of the NWs were investigated using this MOS structure.

Interestingly, Ge NWs could also form under SET operation of the resistive switching memory in an IrO_*x*_/GeO_*x*_/W MIM structure. Oxygen ion migration and nanofilament (or NW) diameter were also investigated using this MIM structure. Resistive switching memory devices were fabricated on 8-in. Si substrates. A 100-nm-thick W bottom electrode (BE) was deposited by rf magnetron sputtering. To define an active area, a 150-nm-thick SiO_2_ layer was deposited onto the BE. Standard lithography and etching processes were used to expose the active area. Then, a Ge layer with a thickness of 20 nm was deposited from a Ge target by the sputtering method described above. Ar with a flow rate of 25 sccm was used as a sputtering gas during deposition. The deposition power and time were 50 W and 3 min, respectively. An IrO_*x*_ TE of approximately 100 nm was then deposited using an Ir target as outlined above. After a lift-off process, the final MIM resistive switching memory device with a size of 8 × 8 μm^2^ was obtained. Memory characteristics were measured using an LCR meter (HP 4285A, Palo Alto, CA, USA) and semiconductor parameter analyzer (Agilent 4156C, Santa Clara, CA, USA).

## Results and discussion

Figure 2 shows the XPS of Ge/GeO_*x*_ NWs grown by the VLS method. The peaks from the Ge 3*d* core-level electrons were fitted using Gaussian functions. The binding energies of the Ge 3*d* core-level electrons are centered at 29.3 and 32.8 eV, which are related to unoxidized germanium and oxidized germanium, respectively [40]. The peak ratio of GeO_2_/Ge is approximately 1:0.13. The binding energies of the Ge 2*p* core-level electrons were 1,218 and 1,220.4 eV (not shown here). The shift of the Ge 2*p* binding energies indicates the formation of Ge suboxides [40]. This suggests that Ge/GeO_*x*_ layers are observed rather than pure Ge NWs, which should help to obtain good resistive switching memory characteristics.

To observe the defects in the Ge/GeO_*x*_ NWs, we recorded PL spectra of the NWs, as shown in Figure 3a. To understand the temperature dependence of the PL spectra, the peak was normalized with respect to PL at 300 K. No significant shift of the emission peak with temperature was observed. However, the PL intensity gradually increases as the temperature increases from 10 to 300 K, revealing that more defect states are activated as the temperature is raised. To identify the defects inside the Ge/GeO_*x*_ NWs, the PL spectrum measured at 300 K was decomposed into four component peaks using Gaussian fitting, as shown in Figure 3b. The peaks are centered around 387 nm (3.2 eV), 402 nm (3.1 eV), 433 nm (2.9 eV), and 483 nm (2.6 eV). Violet-blue emission is observed from these Ge/GeO_*x*_ NWs. Because of their large diameter of approximately 100 nm, the quantum confinement effect is not the origin of this broad emission spectrum [41]. Therefore, the PL peaks probably originate from oxygen vacancies (*V*_o_), oxygen-germanium vacancy pairs (*V*_Ge_, *V*_o_), and related defects. The broad violet-blue emission can be explained by a simple mechanism. It is assumed that acceptors will form (*V*_Ge_, *V*_o_), and the donors will form *V*_o_. After the excitation of acceptors/donors, a hole (*h*_o_) and electron (*e*) are created on the acceptor and donor, respectively, forming (*V*_Ge_, *V*_o_) and (*V*_o_) according to the following equation [42]:

(1) $${\left(V_{\mathrm{Ge}},V_{o}\right)}^{+}+{\left(V_{o}\right)}^{-}+2\mathrm{h\nu}=\left(V_{\mathrm{Ge}},V_{o}\right)+\left(V_{o}\right)+h_{o}+e,$$

where *h* is Plank's constant and *ν* is frequency. The violet-blue emission occurs via the reverse reaction. This suggests that the vacancies exist in the Ge/GeO_*x*_ NWs, which may improve their resistive switching memory performance.

A schematic diagram of the NW-embedded MOS capacitor in an IrO_*x*_/Al_2_O_3_/Ge NWs/p-Si structure is shown in Figure 4a. The capacitance (*C*)-voltage (*V*) hysteresis characteristics of the Ge/GeO_*x*_ NW capacitors with different sweeping voltages from ±1 to ±5 V were investigated, as shown in Figure 4b. Memory windows of 1.7 and 3.1 V are observed under small sweeping gate voltages of ±3 and ±5 V, respectively. In contrast, a small memory window of 1.2 V under a sweeping gate voltage of ±7 V was observed for the device without Ge/GeO_*x*_ NW capacitors because of the degradation of the GeO_*x*_ film (data not shown here). The larger memory window of the device containing Ge/GeO_*x*_ NW capacitors compared with those without the capacitors may be caused by effective charge trapping on the surface of the Ge/GeO_*x*_ NWs. Defects on the surface of the Ge/GeO_*x*_ NWs will trap holes rather than electrons because the *C*-*V* signal shifted towards the negative side, which was also observed in the PL spectrum of the NWs. Applying a larger gate voltage of >5 V caused the MOS capacitor to degrade because of conducting path formation or soft breakdown caused by the Ge-O bonds on the Ge/GeO_*x*_ NW surface breaking. Generally, Ge-O bonds are weakened as the number of oxygen vacancies increases. Figure 4c shows typical *I*-*V* switching characteristics of a Ge/GeO_*x*_ NW capacitor. By applying a positive voltage to the IrO_*x*_ TE, oxygen ions move as a negative charge towards the Al_2_O_3_ layer and set the device at high current (SET) (the low resistance state (LRS)). By applying a negative voltage to the IrO_*x*_ TE, oxygen ions move towards the surface of the Ge/GeO_*x*_ NWs and oxidize the conducting path, which resets the device to low current (RESET) (the high resistance state (HRS)). The resistive switching mechanism of the MIM structure is explained later. Large SET and RESET voltages of +5.1 and −4.0 V, respectively, were found. The oxidation states of the materials in a MOS structure can be explained in terms of Gibbs free energy. The Gibbs free energies of IrO_2_, SiO_2_, Al_2_O_3_, and GeO_2_ at 300 K are −183.75, −853.13, −1,582.3, and −518.5 kJ/mol, respectively [43]. This suggests that IrO_2_ or IrO_*x*_ is an inert electrode. However, the Al_2_O_3_ and SiO_2_ films will oxidize more easily than the GeO_2_ film. Therefore, both SiO_2_ and Al_2_O_3_ layers will insulate the surface of the NWs. The AlO_*x*_ layer will take more oxygen from GeO_*x*_/Ge NW surface. Then, the Ge NW surface will be more defective, and it is also thicker than Al_2_O_3_ (100 vs. 10 nm), which is reasonable to form the conducting filament through the Ge/GeO_*x*_ NW surface rather than the filament formation in the Al_2_O_3_ film. The current passing through the NW surface will therefore be self-limited because of the insulating layers (SiO_2_ and Al_2_O_3_) and also the large diameter (approximately 100 nm) of the Ge NWs (i.e., long conducting pathway). As a result, the resistive switching memory of this device with a MOS structure has a low current compliance (CC) of <20 μA. Similar self-controlled current limitation caused by a series resistance effect has been reported previously [25,34]. A high resistance ratio (HRS/LRS) of approximately 10^4^ is observed at a read voltage of +2 V. However, after few cycles, the resistance ratio is reduced to approximately <10. This may be related to the large gate area of 3.14 × 10^−4^ cm^2^, which makes it difficult to control conducting path formation/rupture between cycles. Therefore, a small device is needed to control the repeatable SET/RESET switching cycles. Figure 4d shows the data retention characteristics of the Ge/GeO_*x*_ NW capacitors. The memory device with a resistance ratio (HRS/LRS) of approximately 2 has good data retention of 2,000 s, which is suitable for use in nanoscale low-power nonvolatile memory applications. A Ge/GeO_*x*_ NW resistive switching memory device can also be formed in an IrO_*x*_/GeO_*x*_/W structure under external bias, which is explained in detail below.

**Figure 5:**
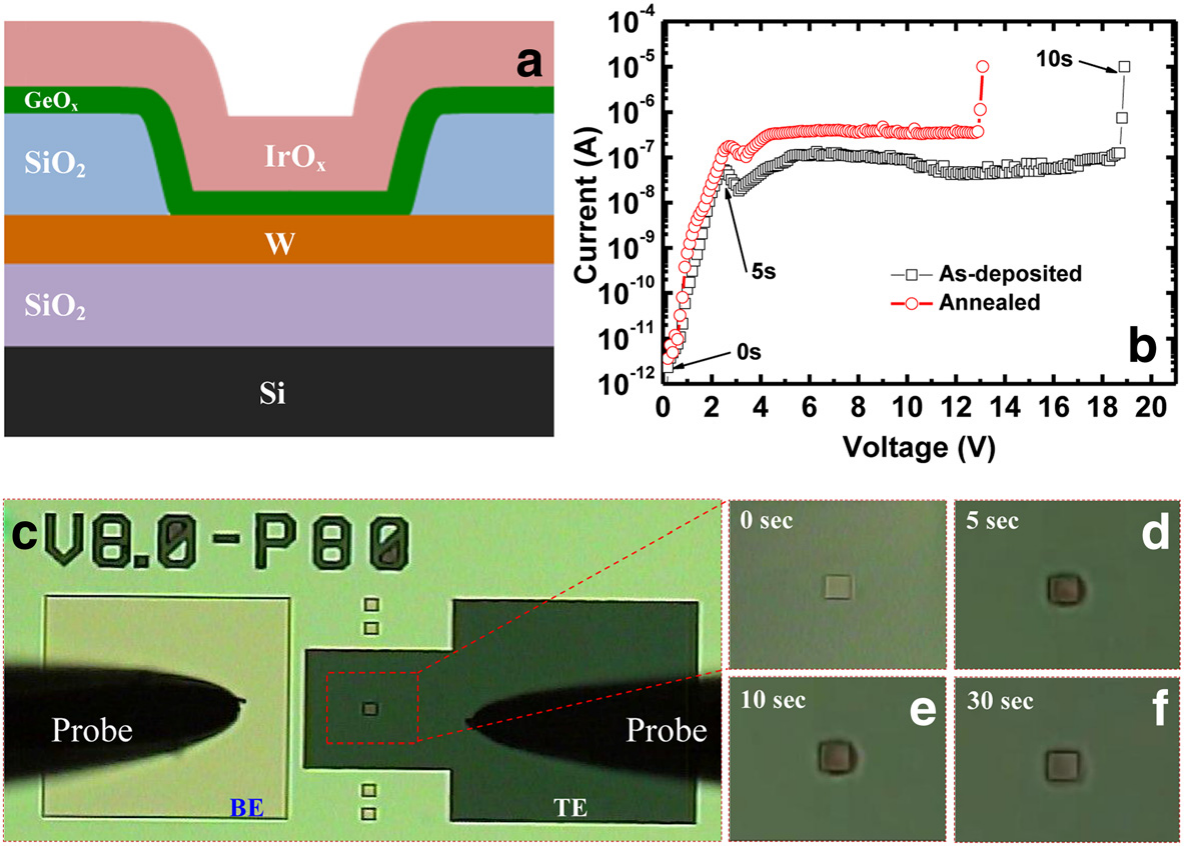
**IrO**_***x***_**/GeO**_***x***_**/W MIM structure, typical *I*-*V *characteristics, and migration of oxygen ions.** (**a**) Schematic diagram of the IrO_*x*_/GeO_*x*_/W MIM structure. (**b**) Typical *I*-*V* characteristics of as-deposited and PMA devices. (**c** to **f**) The migration of oxygen ions during application of a formation voltage, as shown in (**b**).

**Figure 6:**
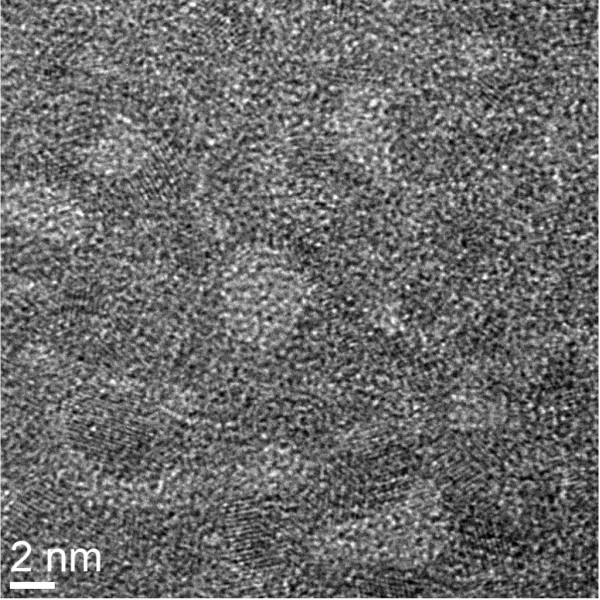
**Plan-view TEM image of an IrO**_***x ***_**layer.** With a typical thickness of approximately 3 nm on the SiO_2_/Si substrate. The IrO_*x*_ metal is black and SiO_2_ is white. The IrO_*x*_ metal layer contains pores that oxygen can readily migrate through.

Resistive switching memory using an IrO_*x*_/GeO_*x*_/W MIM structure is shown in Figure 5a. To obtain typical bipolar hysteresis characteristics, a large formation voltage of approximately 19 V is initially applied to the as-deposited devices (Figure 5b). However, the formation voltage is reduced to approximately 13 V after PMA treatment of the device at 400°C for 10 min under N_2_. The leakage currents of the as-deposited and annealed devices are 1.2 × 10^−10^ and 7.5 × 10^−10^ A, respectively, at a read voltage (*V*_read_) of +1 V. This suggests that Ge-O bonds are volatized [42], and more oxygen vacancies are created after annealing. It is known that the melting points of Ir, IrO_2_, Ge, and GeO_2_ are 2,466°C, 1,100°C, 937.4°C, and 1,115°C, respectively. The annealing temperature (400°C) is much lower than the melting points of the above materials. Therefore, the interdiffusion between IrO_*x*_ and GeO_*x*_ layers is not possible. However, the outdiffusion of oxygen from GeO_*x*_ layer happened after PMA, which results in more leakage pathways through the GeO_*x*_ film. The current conduction pathways are created during the formation process, so resistive switching occurs. These pathways are formed by oxygen ion migration, which was observed *in situ* on the TE surface by optical imaging (OM) during measurement of the device under positive bias. Several static images were obtained from video or real-time observation as the voltage was increased from 0 to 19 V; these are presented in Figure 5c,f. For simplicity, we have given the time scale on the *I*-*V* curve (Figure 5b) and the corresponding static OM images from video as well. Figure 5c shows an OM image of the device surface at time zero (*t* = 0 s) or pristine one. At *t* = 5 s, the current increases, and the device surface is partially changed by the evolution of O_2_ gas (Figure 5d). One can see clearly different views on the device active regions between fresh and after 5 s of stress. Black smoke on the active device region is obviously O_2_ gas; however, those are not images during device burning. Our microscope does not have a good resolution. After the formation, the devices showed resistive switching, which proves that O_2_ gas came out indirectly. Under an external electric field, the Ge-O bonds in the GeO_*x*_ film break and O_2_ gas forms. The Ge-O bond breaking process is completed by *t* = 10 s or at the formation voltage, as shown in Figure 5e. After 30 s, there are no O_2_ bubbles (Figure 5f). However, the TE surface has changed, which suggests that the GeO_*x*_ switching material is modified. It is interesting to note that the O_2_ bubbles readily come out through the TE because of the good porosity of the IrO_*x*_ film, as shown in Figure 6. The typical thickness of the IrO_*x*_ film deposited on the SiO_2_ surface was 3 nm. A plan-view TEM image shows a net-type crystalline IrO_*x*_ film (black) on the SiO_2_ surface (white). Under positive voltage on the TE for a fresh device, evolution of O_2_ gas is observed. However, no gas is observed when a negative voltage is applied to the TE. This suggests that the oxygen ions migrate as a negative charge towards the BE, which acts as a sink. This increases the resistivity of the BE, which results in no stable switching phenomena under negative formation voltage on the TE. To obtain resistive switching characteristics, a positive formation process is used in this study. The same resistive switching mechanism also applies for the MOS structure; however, evolution of O_2_ gas was not observed because of the very low current (<20 μA) operation caused by its self-limitation. Overall, the migration of oxygen ions leads to the high current state as well as the resistive switching mechanism for both the MOS and MIM structures.

**Figure 7:**
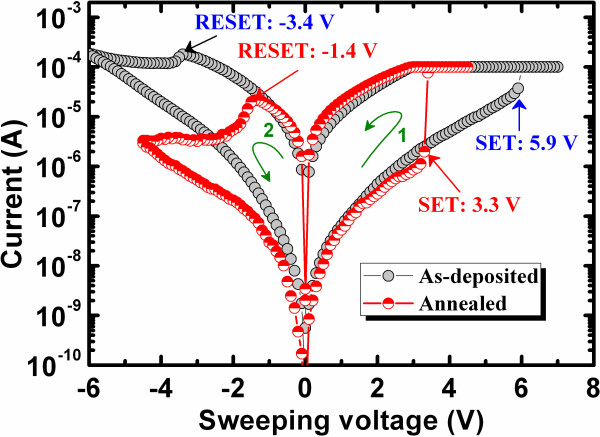
**Typical *I*-*V *hysteresis characteristics of as-deposited and PMA devices with an IrO**_***x***_**/GeO**_***x***_**/W structure.**

**Figure 8:**
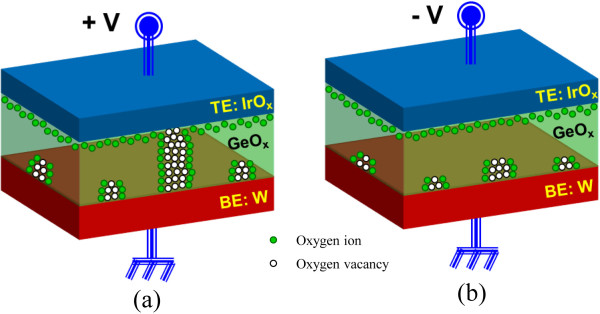
**Formation (a) and oxidation (b) of Ge/GeO_*x *_nanofilaments under SET and RESET operations.** Ge/GeO_*x *_nanowires can be formed under SET and it is dissolved under RESET operations.

Typical *I*-*V* hysteresis characteristics for the as-deposited and PMA devices are presented in Figure 7. A low CC of 100 μA was observed. The SET/RESET voltages were +5.9/−3.4 V and +3.3/−1.4 V for the as-deposited and PMA devices, respectively. The RESET current of the PMA device is lower than the CC (approximately 22 μA) because there is no parasitic effect [44], which has also been observed in a MOS structure (Figure 4c). The PMA device exhibits lower operating current and SET/RESET voltages because PMA increases the number of oxygen vacancies. Furthermore, the resistance ratio (1,750 vs. 408) is also increased after PMA, which may be related to the larger diameter of the filaments. After the formation and first RESET, the device could be consecutively switched between LRS and HRS by applying SET and RESET voltages, respectively, to the TE. Under SET voltage, the O^2−^ ions migrate towards the TE and form an oxygen-rich GeO_*x*_ layer (i.e., GeO_2_) at the GeO_*x*_/TE interface, as shown in Figure 8a. However, the evolution of O_2_ gas is not observed under SET voltage because of the small amount of oxygen present. When the Ge-O bonds break, Ge-rich GeO_*x*_ nanofilaments or Ge/GeO_*x*_ NWs are formed in the GeO_*x*_ bulk material, which will convert the device to the LRS. This suggests that the inside of the filament is Ge-rich and the outside of the filament is oxygen-rich, i.e., a core-shell structure. At RESET voltage, O^2−^ ions will move from the oxygen-rich GeO_*x*_ layer and oxidize the Ge nanofilament, as shown in Figure 8b. The Ge nanofilament is not fully oxidized, and part of the filament remains, which is confirmed by observed leakage current. The leakage currents at *V*_read_ of +1 V are 7.5 × 10^−10^ and 5.1 × 10^−8^ A for a fresh device and that after first RESET, respectively. The higher leakage current after RESET is related to the remaining filament at the GeO_*x*_/W interface. The formation and oxidation of the core-shell Ge/GeO_*x*_ nanofilament by external bias leads to the resistive switching characteristics.

**Figure 9:**
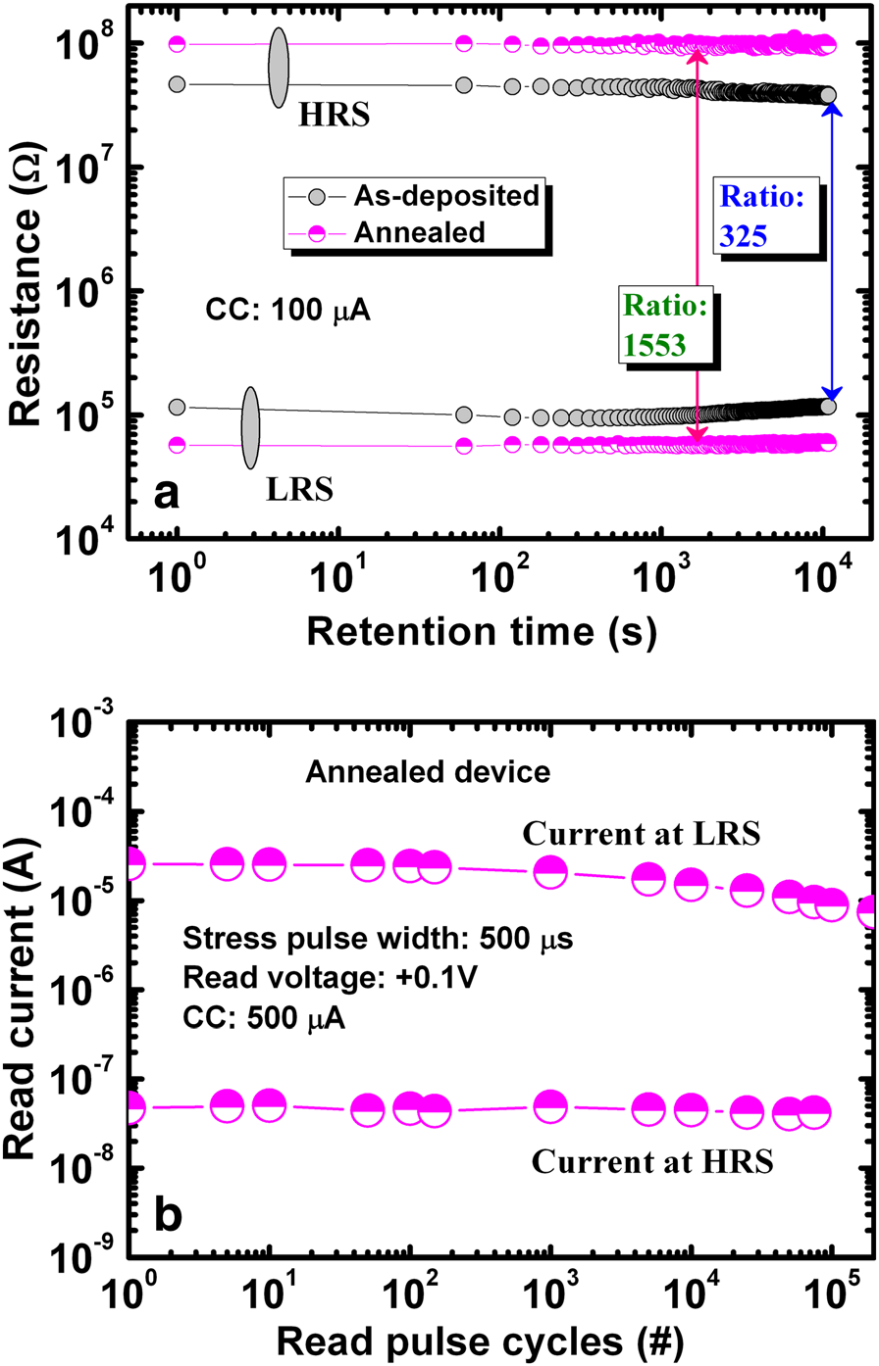
**Data retention characteristics and good pulse read endurance.** (**a**) Data retention characteristics of the IrO_*x*_/GeO_*x*_/W devices. The resistance ratio is larger for the PMA devices than that of the as-deposited one after 10^4^ s. (**b**) Good pulse read endurance of >10^5^ cycles is obtained for the PMA devices.

Figure 9a shows that the IrO_*x*_/GeO_*x*_/W memory devices possess good data retention characteristics before and after annealing under a low CC of 100 μA. Initially, the LRS and HRS values are 57 kΩ and 97.9 MΩ for the PMA device, respectively, whereas they are 115.7 kΩ and 46.2 MΩ for the as-deposited device, respectively. After 10^4^ s, the LRS and HRS values of the PMA device are almost the same (60.2 kΩ and 93.5 MΩ, respectively), whereas the LRS of the as-deposited device is almost the same (116.5 kΩ) but the HRS decreases (37.8 MΩ). Therefore, the resistance ratio losses after 10^4^ s are 18.5% (399 to 325) and 9.5% (1,717 to 1,553) for the as-deposited and PMA devices, respectively. After applying a program/erase current of 500 μA, a long read endurance of >10^5^ cycles with a stress pulse of 500 μs and a read voltage of 0.1V is obtained, as shown in Figure 9b.

**Figure 10:**
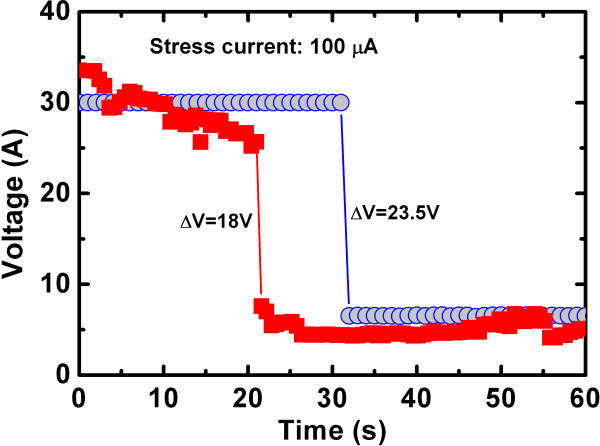
**Evolution of voltage shift under constant current stress on the MIM structure.** The voltage shift is caused by the filament or NW formation in the GeO_*x *_film.

The PMA device shows better performance than that of the as-deposited device, which makes it suitable for nanoscale nonvolatile memory applications. The diameter of the nanofilament was calculated using a new method for oxide-based RRAM devices as follows. Figure 10 shows the soft breakdown (SBD) of the GeO_*x*_ film by applying constant current stress on the TE. The stress current is 100 μA, and the voltage is monitored with time. The initial voltage is high (30 to 34 V), and this suddenly jumps to a low voltage of 6 to 7.5 V for the device-to-device measurement. Because the external constant current stress changes the GeO_*x*_ film from insulating to the defect-rich layer or conducting by Ge-O bond breaking, the voltage across the GeO_*x*_ film is reduced. Due to this Ge-O bond breaking, the conducting path or filament is formed, the current passes easily, and the voltage across the film drops. By observing the voltage drop, it is confirmed that the conducting filament is formed. Definitely, high current stress is not for resistive switching because of strong conducting path formation, which is hard to do RESET operation. By the capture and emission of electrons at an oxide trap inside the GeO_*x*_ film, voltage shifts (Δ*V*_i_) of 18 to 23.5 V are observed. The capture of such electrons can cause a change of trapped charge (Δ*Q* = *ne*) as well as a shift of local injection field (Δ*E*_L_) [45,46]:

(2) $$\Delta Q=C\cdot \Delta V=\left(\frac{\epsilon_{{\mathrm{GeO}}_{2}}.\Phi }{t_{{\mathrm{GeO}}_{2}}}\right)\Delta V,$$

(3) $$\Delta E_{L}=\frac{\Delta V}{t_{{\mathrm{GeO}}_{2}}}=\frac{\mathrm{ne}}{\epsilon_{{\mathrm{GeO}}_{2}}.\Phi },$$

where *e* is the electronic charge (1.602 × 10^−19^ C), *n* is the number of electrons captured, *C* is the capacitance of the MIM capacitor, $\epsilon_{\mathrm{Ge}O_{2}}$ is the dielectric permittivity of the GeO_2_ film (approximately 6 [47]), $t_{\mathrm{Ge}O_{2}}$ is the thickness of the GeO_*x*_ film (approximately 20 nm), and *Ф* is the capture cross-sectional area or the effective area of the conducting paths (nanofilament). Δ*V* is the voltage shift for capturing one electron and is approximately 1 V for the gate oxide (SiO_2_) with a thickness of 4.5 nm [46]. However, the voltage shifts are 18 to 23.5 V, so the total number of electrons captured in the GeO_*x*_ film after SBD is 18 to 23. The cross-sectional area of the cylindrical conducting filament in the GeO_*x*_ film can be expressed as follows:

(4) $$\Phi =\frac{\pi D^{2}}{4},$$

where *D* is the diameter of the nanofilament or NW. Considering Equations 2, 3, and 4, the diameter of the nanofilament is as follows:

(5) $$D=\sqrt{\frac{4\cdot \mathrm{ne}\cdot t_{{\mathrm{GeO}}_{2}}}{\pi \cdot \epsilon_{{\mathrm{GeO}}_{2}}\cdot \Delta V}}$$

and is found to be 37 to 42 nm under an operating current of 100 μA. The diameter can be reduced by decreasing the CC, particularly in the MOS structure (CC < 20 μA). In the case of CBRAM devices, many researchers have reported filament diameters using different materials as well as structures [17,48-50]. Rosezin et al. [48] reported a filament diameter of approximately 13.5 nm at a CC of 100 μA. Liu et al. [17,49] reported a filament diameter of 20 nm with a CC of 1 mA. Yang et al. [50] reported a diameter of 20 nm at a low CC of 10 nA. However, the diameter investigated in this study is different from the reported values, which may be related to the different structure and materials. It is expected that this new method to calculate the diameter of defect paths in oxide-based resistive switching memory devices will be useful in the future.

## Conclusions

Core-shell Ge/GeO_*x*_ NWs were prepared by the VLS technique on Au NP-coated Si substrate. Germanium-oxygen and oxygen vacancies, observed by XPS and broad PL spectra at 10 to 300 K, resulted in good resistive switching memory characteristics of the Ge/GeO_*x*_ NWs in a MOS structure with a low self-compliance of <20 μA. Real-time observation of oxygen ion migration through a porous TE in an IrO_*x*_/GeO_*x*_/W structure and evolution of O_2_ gas during filament formation provided evidence for the resistive switching mechanism. Enhanced memory characteristics such as low-voltage operation (<4 V), low RESET current (approximately 22 μA), large resistance ratio (>10^3^), pulse read endurance of >10^5^ cycles, and data retention of >10^4^ s were obtained for PMA devices because of its volatized nature and the ready formation of oxygen vacancies in the GeO_*x*_ film. Furthermore, a nanofilament diameter of approximately 40 nm in the RRAM device was calculated using a new method. Overall, the properties of this memory device suggest that it is suitable for nanoscale nonvolatile memory applications.

## Competing interests

The authors declare that they have no competing interests.

## Authors’ contributions

AP fabricated and measured the devices under the instruction of SM (Siddheswar Maikap). SZR also helped to fabricate MIM device and measurement under the instruction of SM (Siddheswar Maikap). SM (Sandip Majumdar) and SM (Santanu Manna) fabricated Ge NWs and measured PL spectra under the instruction of SKR. All the authors contributed to the revision of the manuscript, and they approved it for publication. All authors read and approved the final manuscript.
